# A relay strategy for the mercury (II) chemodosimeter with ultra-sensitivity as test strips

**DOI:** 10.1038/srep15987

**Published:** 2015-11-06

**Authors:** Zhijun Ruan, Conggang Li, Jian-Rong Li, Jingui Qin, Zhen Li

**Affiliations:** 1 Department of Chemistry, Hubei Key Lab on Organic and Polymeric Opto-Electronic Materials, Wuhan University, Wuhan 430072, China; 2 China State Key Laboratory of Magnetic Resonance and Atomic and Molecular Physics, Wuhan Institute of Physics and Mathematics, The Chinese Academy of Sciences, Wuhan, 430071, China; 3 Department of Chemistry and Chemical Engineering, Beijing University of Technology, Beijing, 100124, China

## Abstract

A relay strategy has been proposed to design a new Hg^2+^ chemodosimeter (**TPE-S**), by coupling Hg^2+^-promoted deprotection reaction with ketone-enol isomerization, realizing the multistage amplifying effect. Changes in both of color and fluorescence could occur immediately, and **TPE-S** displayed high selectivity for Hg^2+^, other metal ions (Ag^+^, Fe^3+^, Cu^2+^, Pb^2+^, Co^2+^, Cr^3+^, Al^3+^, Cd^2+^, Mg^2+^, Mn^2+^, Ba^2+^, Fe^2+^, Ca^2+^, Ni^2+^, Zn^2+^, Li^+^, K^+^ and Na^+^) gave nearly no disturbance to the sensing process. When fabricated as test strips similar to pH-indicator papers, immediate color change from colorless to purple could be visually observed by naked-eyes without the aid of any additional equipment, with the detection limit as low as 1 × 10^−7^ M (Hg^2+^ in aqueous solution). Due to its easy synthesis, high selectivity and sensitivity, combined with the portable test strips, **TPE-S** could be developed as a convenient and cost-effective tool for the detection of Hg^2+^ in on-site inspections.

Nowadays, diverse sensors and/or probes are badly needed, to monitor the environment pollution, ensure the food safety, and aid the disease surveillance, accompanying with the development of our society and the increasing care on human health^1^,^2^,^3^,^4^,^5^. Among them, sensors based on the chemical reactions attract more and more interests for their high selectivity^6^,^7^,^8^,^9^,^10^,^11^,^12^,^13^. However, the design ideas are straightforward, frequently limited to the conversion of one group to another in consistent with one special reaction. This, somehow, leads to some inherent deficiencies, i.e. relatively low sensitivity. If, the multistage amplifying effect (MAE), realized by more than one step reaction, could be achieved, the designed chemodosimeters would possess better performance, just like the work mechanism of a triode.

Mercury ion, one of the toxic species distributed in air, water and soil, can cause permanent damage to our central nervous and endocrine system^14^,^15^,^16^,^17^,^18^. In addition to the various sensors/probes, we have designed some fluorescent and colorimetric chemodosimeters toward mercury ions, based on the Hg^2+^-promoted deprotection reaction of mercaptal, which demonstrated high selectivity, however, relatively low sensitivity^19^,^20^,^21^. Although the detection limit in solution could be as low as 10 nM, in solid state as test strips, it could only report the presence of mercury ions with the concentration higher than 100 *μ*M, restricted by the reaction itself. Since the real applications to practical samples prefer the usage of probes as test strips, but not in solution, the detection limit should be improved. Then, is it possible to attempt the idea of MAE?

Any textbook of organic chemistry demonstrates that the ketone/enol equilibrium depends on the reactivity of the base and the presence of stabilizing groups. Fortunately, with careful design, some enols are purple, while their corresponding ketones white or pale yellow^22^. Thus, this could be considered as another kind of signal transformation. Once coupled with the Hg^2+^-promoted deprotection reaction, our previous signal from the conversion of mercaptal to ketone might be amplified, possibly to realize the MAE idea.

Prompted by the above thoughts, **TPE-S** was prepared (Fig. 1), which converted to the ketone triggered by trace mercury ions; then, thanks to the presence of nitro group as strong acceptor, chemical base can aid the formation of the corresponding enol with the special D-π-A structure, accompanying with the apparent color change from pale yellow to deep purple. As the result, the detection limit of Hg^2+^ was improved to 0.1 *μ*M as test strips, 1000 times that our previous ones. That is to say, by utilizing the deprotection reaction of mercaptal and the transformation between Ketone and enol, according to the relay strategy, the signal was enlarged 1000 times, confirming the above MAE idea. Herein, we present the synthesis, sensing behavior, and related mechanism in detail.

## Results

### Synthesis and Structural Characterization

As shown in Figure S1 (supplementary information), **TPE-S** was conveniently prepared, in which two saturated carbon atoms (one is the protected ketone) were elaborately designed between the tetraphenylethylene (TPE) and nitrobenzene groups. While the nitrobenzene acting as the acceptor for the followed formed D-π-A structure, the TPE one was a weak donor but with the characteristic of Aggregation Induced Emission (AIE), to endow the possible strong fluorescence in the solid state toward Hg^2+^. Actually, after the first report of AIE phenomenon (molecules nonemissive in solution, but highly emissive once aggregated either in the solid state or as nanoparticles) by Tang and coworkers, the research on AIE becomes a hot topic, with TPE considered as the star AIE molecule^23^,^24^,^25^,^26^,^27^,^28^,^29^. For comparison, **TPE-1** and **TPE-2** without the nitro group, were also synthesized. All compounds were well characterized by ^1^H and ^13^C NMR (supplementary Figure S20–27), mass spectrometry, Fourier transform infrared spectra, elemental analysis, and X-ray crystallography (supplementary Figure S4-5 and Table S1).

### AIE Properties

**Figure 2:**
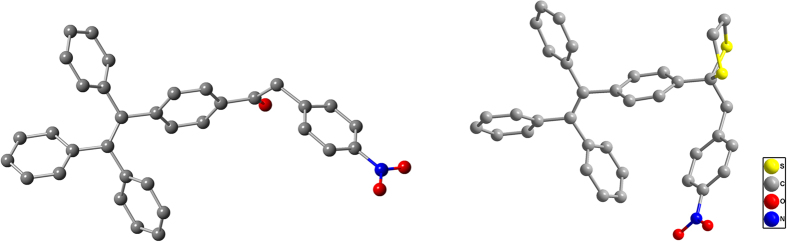
Crystal structures of TPE-O (left) and TPE-S (right). The nitro group in **TPE-O** was far from the TPE group, while the nitro one in **TPE-S** was close to the TPE one. The gray balls represent C, the red balls represent O, the blue balls represent N, the yellow balls represent S, hydrogen were omitted for clean. The crystal packing structures and the corresponding data were shown in Figure S4–5 and Table S1 in Supplementary information.

The AIE characteristic was utilized to enhance the fluorescence of the designed probe in the solid state. Thus, photoluminescence (PL) spectra of **TPE-O**, **TPE-S**, **TPE-1** and **TPE-2**, were measured in CH_3_CN-H_2_O mixtures with different water fractions (*f*_w_), to investigate their AIE properties (supplementary Figure S2–3). In CH_3_CN solutions, they were nonemissive. Upon the addition of water, these TPE-containing molecules aggregated into nanoparticles step by step. As a result, their PL intensity increased apparently with the increasing water fraction, when *f*_w_ higher than 70%. As visually shown in the inset photos, **TPE-1** and **TPE-2** were AIE-active, **TPE-O** containing the nitro group, emitted not so strong (*f*_w_ = 95%), but still possessing AIE characteristic, while **TPE-S** not. This was understandable. From the crystal structures of **TPE-O** and **TPE-S** (Fig. 2), it was easily seen that the nitrobenzene moieties in **TPE-S** was highly twisted, so the nitro group was close to the TPE moiety, most probably quenching its possible emission in the solid state. However, in **TPE-O**, due to the sp^2^-hybrid conformation of the ketone carbon (sp^3^ in **TPE-S**), the nitro group was much far from the TPE moiety, weakening the quenching effect. Then, once triggered by Hg^2+^, the conversion of **TPE-S** (weak fluorescence intensity in aggregation state) to **TPE-O** (high fluorescence intensity in aggregation state) could give out the fluorescent signal in the solid state, to report the presence of Hg^2+^.

### Hg^2+^ Sensing Properties

**Figure 3:**
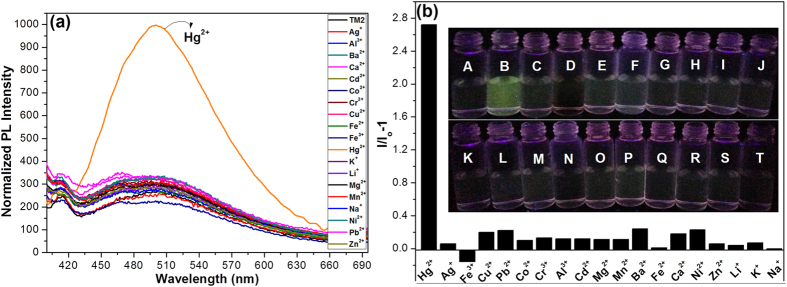
Fluorescence turn-on probe for the detection of Hg^2+^. **(a**) Normalized emission spectra of **TPE-S** (20 *μ*M) in the presence of various metal ions ([Hg^2+^], 3 × 10^−4^ M; other ions, 6 × 10^−4^ M) excited at 361 nm in CH_3_CN-H_2_O mixtures with the water fraction of 98%. (**b**) Fluorescence responses of **TPE-S** (20 *μ*M) to various metal ions ([Hg^2+^], 3 × 10^−4^ M; other ions, 6 × 10^−4^ M) excited at 361 nm in CH_3_CN-H_2_O mixtures with the water fraction of 98%. Inset: The corresponding fluorescence photos of **TPE-S** reacted with various metal ions. (**A**) **TPE-S**; (**B**) **TPE-S**+Hg^2+^; (C-T), Ag^+^, Fe^3+^, Cu^2+^, Pb^2+^, Co^2+^, Cr^3+^, Al^3+^, Cd^2+^, Mg^2+^, Mn^2+^, Ba^2+^, Fe^2+^, Ca^2+^, Ni^2+^, Zn^2+^, Li^+^, K^+^, Na^+^.

The sensing process of **TPE-S** was shown in Figure S6 (supplementary information). As **TPE-O** was AIE-active, but **TPE-S** nearly not, thus, based on the Hg^2+^-promoted deprotection reaction, **TPE-S** should be a fluorescence turn-on probe of Hg^2+^. Thus, various metal ions (Hg^2+^, Ag^+^, Fe^3+^, Cu^2+^, Pb^2+^, Co^2+^, Cr^3+^, Al^3+^, Cd^2+^, Mg^2+^, Mn^2+^, Ba^2+^, Fe^2+^, Ca^2+^, Ni^2+^, Zn^2+^, Li^+^, K^+^ and Na^+^) were added to the solution of **TPE-S** in CH_3_CN, then water was added for the formation of aggregation state. Really, as shown in Fig. 3, only Hg^2+^ ions led to the obvious PL enhancement, while others not. And the different sensing behaviors could be visually seen by the naked-eyes with the aid of a normal UV lamp (Fig. 3b). The high selectivity of **TPE-S** toward Hg^2+^ could be further confirmed by the interference experiments. To the solution of **TPE-S** with one of the other metal ions was added Hg^2+^ subsequently, the fluorescence was still turn-on rapidly (supplementary Figure S7), illustrating the nice anti-interference and excellent selectivity of **TPE-S** toward Hg^2+^.

**Figure 4:**
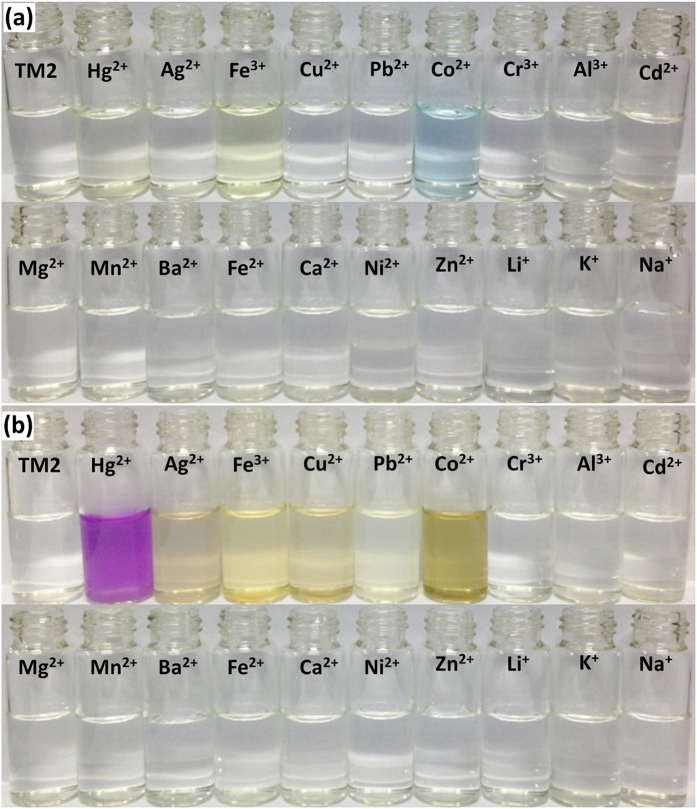
Photos of colorimetric response of TPE-S. (**a**) Photos of colorimetric response of **TPE-S** (20 *μ*M) upon the addition of various metal ions (3 × 10^−4^ M). (**b**) Photos of the solution after 30 equiv. of *t*-BuOK were added to the resultant solution.

Colorimetric sensors have attracted more attention since the color change can be easily observed by the naked-eyes without the need of any equipment. As shown in Fig. 4a and S8 (supplementary information), the dilute solution of **TPE-S** in THF was colorless with maximum absorption wavelength (*λ*_max_) centered at about 245 nm. Upon the addition of Hg^2+^ ions to the solution of **TPE-S**, an obvious change of the UV/Vis spectrum happened when the concentration of Hg^2+^ ions as low as 1 *μ*M. With the increasing concentration of Hg^2+^ ions, the *λ*_max_ shifted from 245 to 232 nm gradually, accompanying with the increasing intensity of the new absorption peak. When the concentration reached to 30 *μ*M, no obvious difference could be observed by further increasing the concentration of Hg^2+^ ions, and the color of the reaction solution changed from colorless to pale yellow (Fig. 4a). In order to compare the reaction solution of **TPE-S** (after adding Hg^2+^) with that of **TPE-O**, and exclude the possible influence from the introduced trace water in the added Hg^2+^ solution, the absorption spectra of **TPE-O** and **TPE-S** were investigated after the addition of trace water and Hg^2+^ ions (supplementary Figure S9). The UV/Vis spectrum of **TPE-O** and **TPE-S** had no changes even the added water was 30 *μ*L. Interestingly, the UV/Vis spectrum of **TPE-O** with and without Hg^2+^ ions were different, indicating that there were some interactions between Hg^2+^ and **TPE-O**. Meanwhile, the UV/Vis spectra of **TPE-S** and **TPE-O** in the presence of Hg^2+^ ions were almost the same, confirming that the Hg^2+^-promoted deprotection reaction of **TPE-S** occurred as expected, and the ketone structure of **TPE-O** was formed. In addition, as shown in Figure S10 (supplementary information), the Hg^2+^-promoted deprotection reaction of **TPE-S** happened immediately. Also, as demonstrated in Figure S8 (supplementary information), there was a good linear relationship between the intensity change and the concentration of Hg^2+^ ions.

Similar to the fluorescent sensing process, other metal ions including Ag^+^, Cu^2+^, Pb^2+^, Cr^3+^, Al^3+^, Cd^2+^, Mg^2+^, Mn^2+^, Ba^2+^, Fe^2+^, Ca^2+^, Ni^2+^, Zn^2+^, Li^+^, K^+^ and Na^+^, did not affect the specific probe toward Hg^2+^ (Fig. 4a and supplementary information Figure S11). However, Fe^3+^ and Co^2+^ ions caused the color change from colorless to yellow and sky blue, respectively. Considering the color of Fe^3+^ solution was yellow, Fe^3+^ ions was added to pure THF, the color changed to yellow as expected, and the UV/Vis spectrum of THF with/without **TPE-S** after the addition of Fe^3+^ were almost the same (supplementary Figure S12), disclosing that Fe^3+^ itself caused the color change. As to the case of Co^2+^, it seemed very confused at the very beginning. Later, thinking that H_2_O could cause the color change of cobalt complexes by change the coordination number of H_2_O^30^, a control experiment was conducted: the aqueous solution of Co^2+^ was added to pure THF, the solution color changed to sky blue immediately; then, 100 μL of H_2_O was added to the blue solution of THF with/without **TPE-S**, the color of the solution returned back to colorless (supplementary Figure S13). Thus, it was speculated that: THF replaced a part of H_2_O coordinated to cobalt ions, causing the color change to sky blue. From the experimental results, it was concluded that the selectivity of the Hg^2+^-promoted deprotection reaction of mercaptal was excellent in both cases of **TPE-S** as fluorescent and colorimetric probe, but the sensitivity was too low and far from the “naked-eye” detection. To solve this problem, a relay strategy was implemented to realize an MAE.

The ketones could be liable to the conversion to enols in the presence of chemical base. Thus, after the addition of different metal ions to the solution of **TPE-S**, *t*-BuOK was added subsequently. Excitedly, the ketone-enol isomerization reaction occurred in the reaction solution of **TPE-S + Hg**^**2+**^ immediately, with an apparent color change from pale yellow to red purple (Fig. 4b), and a new absorption peak (*λ*_max_ = 558 nm) appeared (supplementary Figure S14). However, the solution of **TPE-S** and **TPE-S** with other metal ions nearly remained unchanged, except some light-colored precipitates formed in the solutions containing Ag^+^/Fe^3+^/Cu^2+^/Pb^2+^ or Co^2+^. Therefore, only Hg^2+^ could promote the deprotection reaction of mercaptal very well, and caused the followed ketone-enol isomerization with the aid of *t*-BuOK rapidly. More importantly, the noticeable color change from pale yellow to red purple could be easily observed by naked-eyes, confirming the success of the relay strategy.

### Mechanism of the sensing process

The relay strategy contained two steps: (1) the deprotection reaction of **TPE-S** triggered by Hg^2+^ to form the ketone **TPE-O**; (2) the ketone-enol isomerization in the presence of *t*-BuOK. To confirm the sensing mechanism, control experiments were conducted, in addition to the spectroscopic characterizations.

As shown in Figure S15 (supplementary information), without the presence of the carbonyl group, no corresponding absorption peak at ~1684 cm^−1^ was observed in the IR spectrum of **TPE-S**. After the addition of Hg^2+^ ions, the absorption peak of carbonyl group appeared at 1684 cm^−1^, proving the successful deprotection reaction of **TPE-S**. Also, the main peak at 495.3 (the same to that of **TPE-O)**, in the MS spectrum of the reaction product of **TPE-S** with Hg^2+^, further confirmed the successful deprotection reaction (supplementary Figure S16).

**Figure 5:**
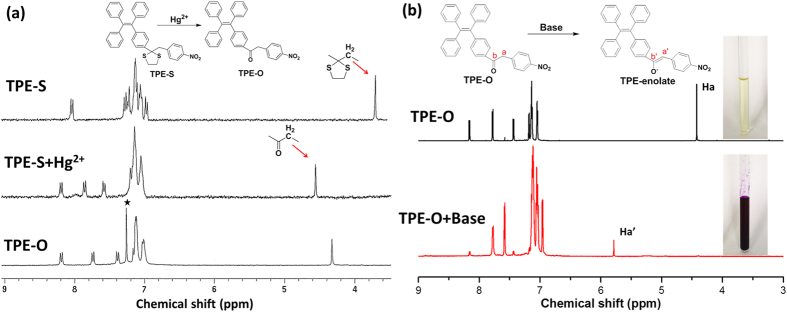
The chemical reaction during the sensing process monitored by NMR. (**a**) ^1^H NMR spectra of **TPE-S** (in acetone-*d*_6_) before and after the addition of Hg^2+^ ions, and **TPE-O** in chloroform-*d*. The solvent peak is marked with asterisk. (**b**) ^1^H NMR spectra of **TPE-O** (in acetonitrile-*d*_3_) before and after the addition of *t*-BuOK. Inset: Photographs of **TPE-O** before and after the addition of *t*-BuOK.

^1^H, ^13^C, and HMBC NMR spectroscopy were used to monitor the chemical reaction during the sensing process. Figure 5a showed the ^1^H NMR spectra of **TPE-S** before and after the addition of Hg^2+^ ions. After the addition of Hg^2+^, the singlet assigned to the methylene shifted from 3.69 to 4.55 ppm, as the result of the formation of carbonyl group. This resultant spectrum was almost the same as that of **TPE-O**, confirming the successful deprotection reaction between **TPE-S** and Hg^2+^. The formation of the enolate after the addition of *t*-BuOK was also proved by ^1^H and ^13^C and HMBC NMR spectroscopy (supplementary Figure S17–18). After *t*-BuOK was added to **TPE-O**, the signal of the methylene (*H*a) attached to the carbonyl group, shifted from 4.43 to 5.79 ppm (*H*a’) (Fig. 5b). Simultaneously, as demonstrated in the inset of Fig. 5b, the color of the solution was changed from pale yellow to purple for the formation of **TPE-enolate**. Meanwhile, in the ^13^C NMP spectra, signals at 45.2 and 196.7 ppm, assigned to the *C*a and *C*b in **TPE-O** respectively, shifted to 94.6 (*C*a’) and 178.1 ppm (*C*b’) upon the formation of **TPE-enolate**. Thus, all these results proved the two steps of the relay strategy: the Hg^2+^-promoted deprotection reaction, and the ketone-enol isomerization.

### Test strips

**Figure 6:**
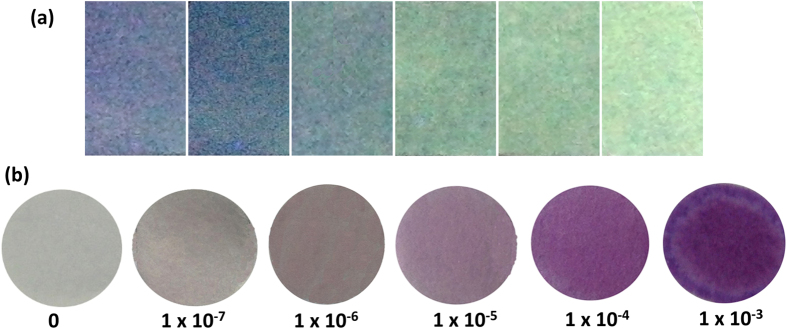
Test strips. (**a**) Photos of fluorescence response of **TPE-S** test strips exposed to different concentrations of Hg^2+^ ions in water, then dried in air. From left to right: single **TPE-S** test strip; **TPE-S** test strips with different concentration of Hg^2+^ ions: 1 × 10^−7^, 1 × 10^−6^, 1 × 10^−5^, 1 × 10^−4^, 1 × 10^−3^ M. (**b**) Photos of colorimetric response of **TPE-S** test strips exposed to different concentrations of Hg^2+^ ions in water, and then dropped some *t*-BuOK (saturated solution in *t*-butanol). From left to right: single **TPE-S** test strip; **TPE-S** test strips with different concentration of Hg^2+^: 1 × 10^−7^, 1 × 10^−6^, 1 × 10^−5^, 1 × 10^−4^, 1 × 10^−3^ M.

Test strips are convenient and cost-effective tool for actual applications. The excellent selectivity and high sensitivity of **TPE-S** prompted us to investigate its practical applications, thus, the test strips were easily fabricated by immersing filter paper into the solution of **TPE-S** and then dried in air. Just as the use of pH-indicator papers, the prepared test strips were utilized to probe the trace Hg^2+^ ions in aqueous solutions. Figure 6a showed the changes of the test papers excited at 365 nm under a UV lamp. Thanks to the AIE effect, the test paper could detect Hg^2+^ at a concentration of about 1.0 × 10^−6^ M, with “turn-on” emission signal. This relatively high detection limit, 100 times the strips in our previous cases^19^,^20^,^21^, demonstrated that the introduction of TPE, the typical AIE luninogen, indeed favored the dramatically fluorescent enhancement in test strips. To further amplify the sensing signal, *t*-BuOK was dropped onto the test strips, which had been immersed into the aqueous solutions of Hg^2+^ ions with different concentrations, immediate color change from colorless to deep purple was observed (Fig. 6b). Excitedly, the discernible concentration of Hg^2+^ ions, by naked-eyes, could be as low as 1.0 × 10^−7^ M (20 ppb). Furthermore, the probe of Hg^2+^ ions in some spiked samples, including tap water and lake water, gave similar results. As the standard of Hg^2+^ ions in the industrial waste water is 50 μg/L (50 ppb), **TPE-S** can be utilized to monitor the concentration of Hg^2+^ ions in the waste water for on-site inspections. Actually, some wonderful works of strip based platforms had reported for the determination of mercury ions with high sensitivity by using enzyme, DNA or oligonucleotides^31^,^32^,^33^. However, since the biomacromolecules were fragile, the strips were unstable for storage and too sensitive to heat, UV radiation, strong acid and base, in addition to their expensive cost, complicated fabrication, and boring test procedure. On contrary, the preparation of our test strips was very simple and cheap (just immersing filter paper into the solution of TPE-S and dried in air), and the usage was very convenient. Moreover, the test strips could be simply stored for a long time just like the normal pH test papers, derived from much higher stability of TPE-S than biomacromolecules (very stable in conventional environment). With the aim to highlight the double response of **TPE-S**, the Hg^2+^ solution were written onto test strips directly, and *t*-BuOK were dropped subsequently. As shown in Figure S19 (supplementary information), rapid changes in the fluorescence and color of the test papers were observed.

## Discussion

Mercury in its various forms has many harmful influences on human body and the surrounding environment. For the present global mercury pollution from nature and human activities, it is urgent for the detection methods with high selectivity and sensitivity, coupled with the features of rapid, convenient and cost-effective^34^,^35^,^36^. Great efforts have been made to develop straightforward methods such as colorimetric, fluorescence or electrochemical changes for the detection of Hg^2+^ by using biomacromolecules^37^,^38^,^39^,^40^,^41^,^42^,^43^, nanoparticles^44^,^45^,^46^,^47^ and different molecules^48^,^49^,^50^,^51^,^52^,^53^. However, a significant bottleneck was the relatively low sensitivity as test strips, although the detection limit in solution could be very good. Actually, for the practical applications, especially for the monitoring of the analytes in food, environment, and even some human body fluid (for example, blood, urine), the calculated detection limit from the experiments conducted in solutions, is, somehow, not so meaningful. On the contrary, those, observed directly by the naked-eyes without the aid of any equipment in solid states, is indeed reliable for the convenient and rapid on-site detections.

Most of the good Hg^2+^ probes suffered the low sensitivity as test strips, partially due to the design strategies. Taking reactive-type Hg^2+^ probes, based on the chemical reactions, as the example, one on hand, the inherent selectivity of the reactions offer the high selectivity of the designed probes, however, on the other hand, the signal derived from the produced molecule depends a little heavily on its quantity, leading to the difficulty in increasing the sensitivity. Thus, perhaps, some new strategies should be developed for the old reactive-type probes. Inspired by the work mechanism of a triode and the cascade reaction, we proposed a relay strategy for the detection of Hg^2+^ with MAE by coupling the Hg^2+^-promoted deprotection reaction with the ketone-enol isomerization. Thus, the sensing signal from the mercaptal to ketone triggered by Hg^2+^, could be boosted from the detection limit of ~10^−4^ to 10^−7^ M as test strips, realizing the effect of “1 and 1 greater than 2”. This might be a new avenue for the further development of reactive-type probes, at least because of the following two points:

1. Some other molecules could be converted to ketone selectively triggered by special species, for example, oxime to ketone in the presence of hypochlorite ions. For this case, the relay strategy could be directly applied by still utilizing the ketone-enol isomerization, to give the signal of color change.
2. Some other types of reactions could be combined together intelligently, to achieving the similar MAE.

In summary, a new Hg^2+^ probe, was successfully designed, based on Hg^2+^-promoted deprotection reaction and ketone-enol isomerization. Thanks to their advantages, including the rapid reaction speed and high selectivity, this proposed relay strategy has been achieved with the ideal MAE. Changes in both of color and fluorescence could happen immediately, and the probe of **TPE-S** displayed high selectivity for Hg^2+^ nearly without disturbance from other metal ions in the sensing process. The fabricated test strips exhibited apparent color change from colorless to purple as visually observed by naked-eye without any additional equipment, with the detection limit as low as 1 × 10^−7^ M (Hg^2+^ in aqueous solution), confirming the practical applications for on-site inspections. Compared to our previous Hg^2+^ probes only based on the Hg^2+^-promoted deprotection reaction with much lower detection limit of ~10^−4^ M as test strips (actually 10 nM in solution, but not meaningful), by applying this relay strategy with the expected MAE, the detection limit of **TPE-S** has been amplified 1000 times for the real applications. Thus, the example of **TPE-S** reported in this paper might be only one tip of the iceberg, and the huge mountain of excellent probes could be developed by applying the proposed relay strategy for on-site inspections.

## Methods

Experimental details, including the synthesis and characterization of compounds, UV-vis spectra, photos and fluorescent spectra, can be found in the Supplementary Information.

### Preparation of the solutions of various metal ions

One millimole of inorganic salt: Hg(ClO_4_)_2_·3H_2_O, AgNO_3_, Al(NO_3_)_3_ 9H_2_O, Cr(NO_3_)_3_·9H_2_O, FeCl_3_·6H_2_O, CoCl_2_·6H_2_O, Ba(NO_3_)_2_, Ca(NO_3_)_2_·4H_2_O, Pb(NO_3_)_2_, Ni(NO_3_)_2_·6H_2_O, Zn(NO_3_)_2_·6H_2_O, MnSO_4_·2H_2_O, Cu(NO_3_)_2_·3H_2_O, Cd(NO_3_)_2_·4H_2_O, Mg(ClO_4_)_2_, Fe(SO_4_)_2_·7H_2_O, NaNO_3_, KNO_3_ and LiCl was dissolved in distilled water (10 mL) to afford 1 × 10^−1^ mol/L aqueous solution, respectively. The stock solutions were diluted to desired concentrations with distilled water when needed.

### Fluorescence intensity changes of TPE-S with different metal ions

A solution of **TPE-S** (1 × 10^−3^ mol/L) in CH_3_CN was prepared. Different metal ions (1 × 10^−1^ mol/L, 9 *μ*L for Hg^2+^ and 18 *μ*L for other ions) were added to the solution of **TPE-S** (60 *μ*L) in a quartz tube respectively, then distilled water was added to help the formation of aggregation state (with water fraction of 98%). The resultant solutions (3 mL) were placed in a quartz cell (10.0 mm width), and the changes of the fluorescence intensity were recorded at room temperature each time (excitation wavelength 361 nm).

### UV-vis titration of TPE-S with Hg^2+^ ions

A dilute solution of **TPE-S** (2.0 × 10^−5^ mol/L) was prepared in THF. The solution of Hg^2+^ (3 × 10^−2^ mol/L) was prepared in distilled water. Solution of **TPE-S** was placed in a quartz cell (10.0 mm width), and the absorption spectrum was recorded. The Hg^2+^ ion solution was introduced in portions, and absorption changes were recorded at room temperature each time.

### UV absorption changes of TPE-S by different metal ions and chemical base

A solution of **TPE-S** (2.0 × 10^−5^ mol/L, 3.0 mL) was placed in a quartz cell (10.0 mm width) and the absorption spectrum was recorded. Different ion solutions (1 × 10^−1^ mol/L, 9 *μ*L) were introduced, and the changes of the absorption changes were recorded at room temperature each time. Then, 30 equiv of *t*-BuOK was added to the resultant solution, and the changes of the absorption changes were recorded at room temperature each time.

## Additional Information

**How to cite this article**: Ruan, Z. *et al.* A relay strategy for the mercury (II) chemodosimeter with ultra-sensitivity as test strips. *Sci. Rep.* **5**, 15987; doi: 10.1038/srep15987 (2015).

## Supplementary Material

Supporting Information

## Figures and Tables

**Figure 1:**
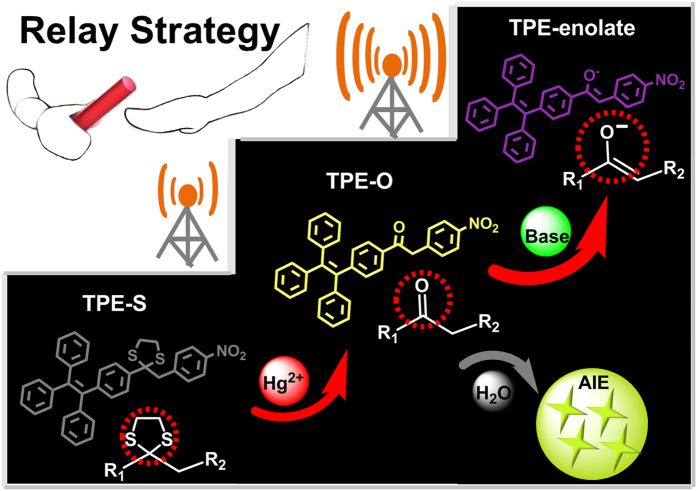
Illustration of the design and the mechanism of the relay strategy. Including the chemical structure and the Hg^2+^ sensing process of **TPE-S** with a multistage amplifying method (this figure was fully drew by Zhijun Ruan).
