# Supplementary material for: A relay strategy for the mercury (II) chemodosimeter with ultra-sensitivity as test strips
Source: Sci Rep. 2015 Nov 6;5:15987. doi: 10.1038/srep15987 (PMC4635349; doi:10.1038/srep15987)
Supplement: Supporting Information [file srep15987-s1.pdf]

Supplementary Information

A relay strategy for the mercury (II) chemodosimeter with ultra-sensitivity as test strips

Zhijun Ruan[1], Conggang Li[2], Jianrong Li[3], Jingui Qin[1] & Zhen Li[1]*

[1]Department of Chemistry, Hubei Key Lab on Organic and Polymeric Opto-Electronic Materials, Wuhan University, Wuhan 430072, China.

Fax and Tel: 86-27-68755363; E-mail: lizhen@whu.edu.cn or lichemlab@163.com.

[2]China State Key Laboratory of Magnetic Resonance and Atomic and Molecular Physics, Wuhan

Institute of Physics and Mathematics, The Chinese Academy of Sciences, Wuhan, 430071, China.

[3]Department of Chemistry and Chemical Engineering, Beijing University of Technology, Beijing, 100124, China

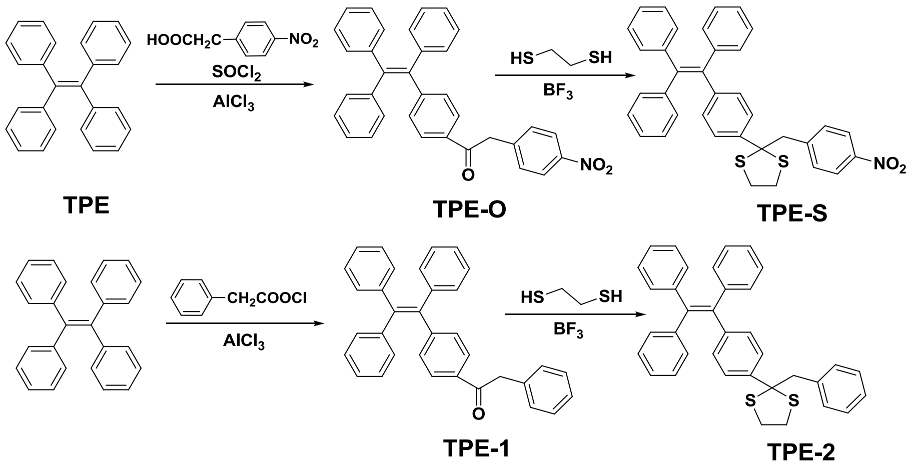

Figure S1. Synthetic way of compounds **TPE-1**, **TPE-2**, **TPE-O** and **TPE-S**.

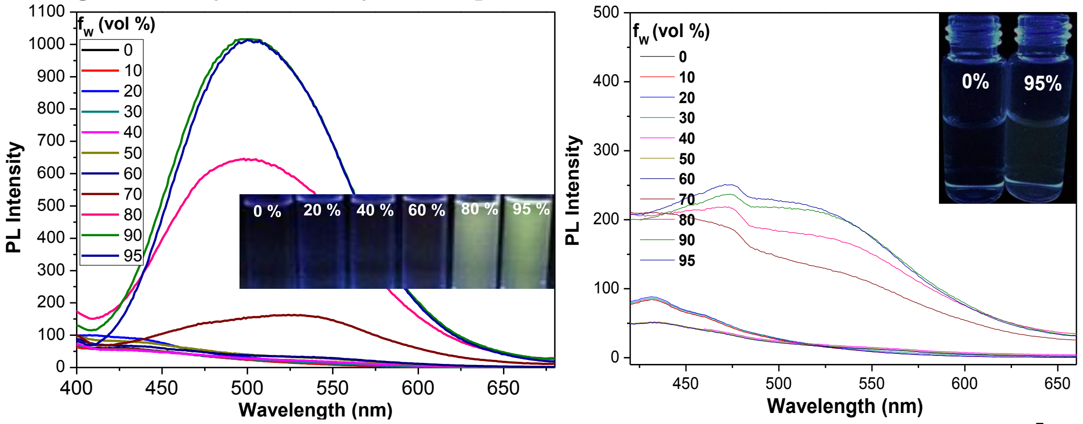

Figure S2. Fluorescence (FL) spectra of **TPE-O** (left) and **TPE-S** (right) (2 x 10^{-5} M) in CH_3CN/water mixtures with different water fractions (excitation/emission slit widths were 5/10 nm, λex: 350 nm). Inset: Fluorescence photograph of **TPE-O** and **TPE-S** at different water fractions.

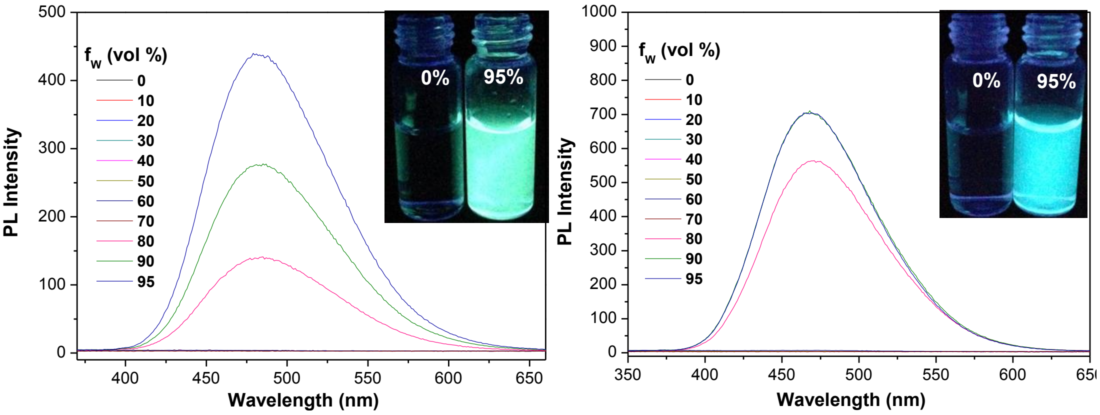

Figure S3. Fluorescence (FL) spectra of **TPE-1** (left) and **TPE-2** (right) (2 x 10^{-5} M) in CH_3CN/water mixtures with different water fractions (excitation/emission slit widths were 1.5/3 nm, λex: 350 nm for **TPE-1** and 330 nm for **TPE-2**). Inset: Fluorescence photograph of **TPE-1** and **TPE-2** at different water fractions.

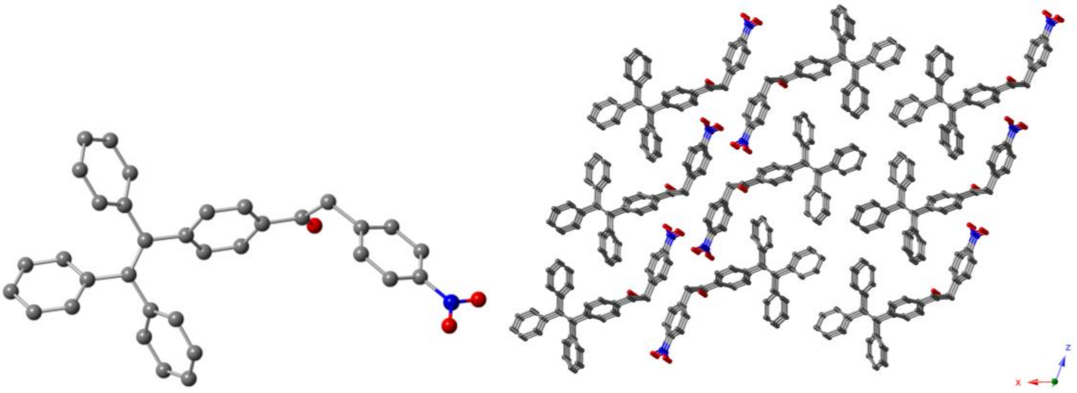

Figure S4. Crystal structure and the crystal packing of **TPE-O**.

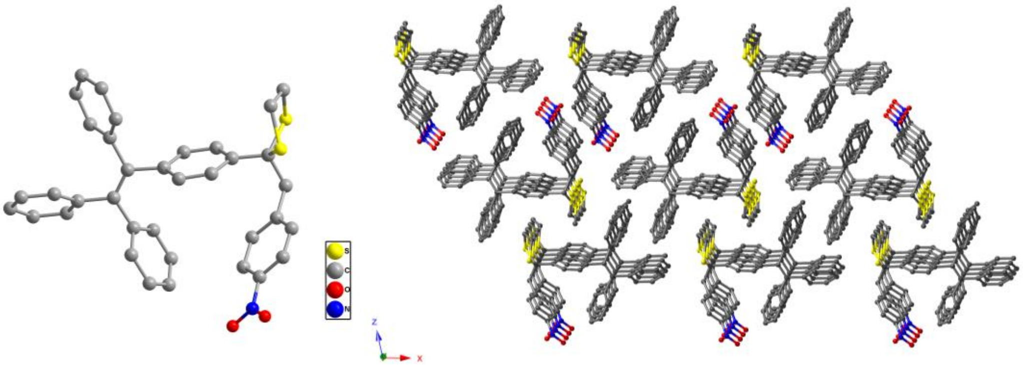

Figure S5. Crystal structure and the crystal packing of **TPE-S**.

Table S1. Summary of crystal data and intensity collection parameters for **TPE-O** and **TPE-S**.

**Compound**	**TPE-O**	**TPE-S**
Formula	$C_{34}H_{25}NO_3$	$C_{36}H_{29}NO_2S_2$
Formula mass	495.55	571.72
Space group	monoclinic, C2/c	monoclinic, P21/n
a/Å	52.118(2)	14.788(3)
b/Å	5.477(2)	6.052(13)
c/Å	19.354(8)	33.853(7)
α/^{o}	90.00	90.00
β/^{o}	110.57(4)	102.21(3)
γ/^{o}	90.00	90.00
V / Å^3	5172.8(4)	2961.4(11)
Z/mg.m^{-3}	8, 1.273	4, 1.282
F000	2080	1200
Theta range/^{o}	1.67 to 26.41	2.07 to 20.29
No. of collected reflns	15048/5146	8061/2804
No. of unique reflns.(Rint)	0.0347	0.0381
Data/restrains/parameters	5146/0/343	2826/0/370
R1,wR2[obs I>2σ(I)]	R1 = 0.0569, wR2 = 0.1366	R1 = 0.0717, wR2 = 0.1979
R1,wR2 (all data)	R1 = 0.0706, wR2 = 0.1497	R1 = 0.0896, wR2 = 0.2163
Residual peak/hole e. Å^{-3}	0.461 and -0.463	0.450 and -0.257
CCDC number	1048022	1048023

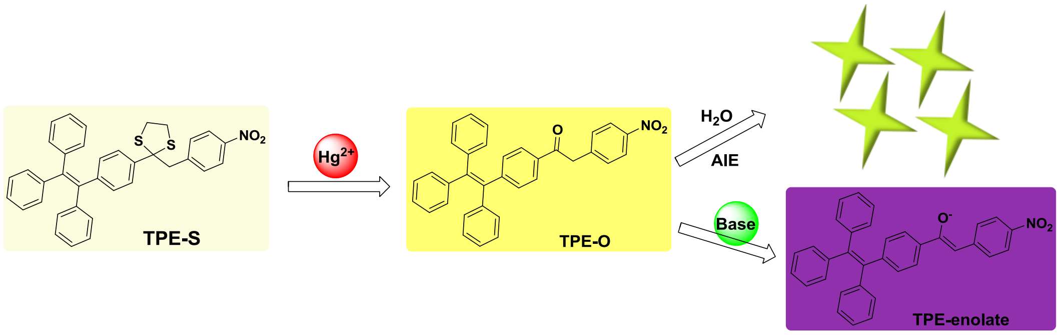

Figure S6. Chemical structure and the Hg^{2+} sensing process of **TPE-S**.

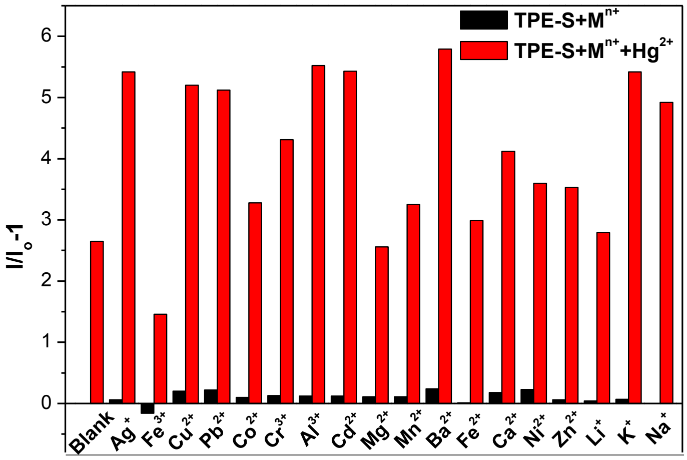

Figure S7. Fluorescence spectra profiles of **TPE-S** (20 μM) in the presence of various metal ions (3 x 10^{-4} M), followed by added Hg^{2+} (3 x 10^{-4} M, red line), in CH_3CN /H_2O (2/98, v/v) solution.

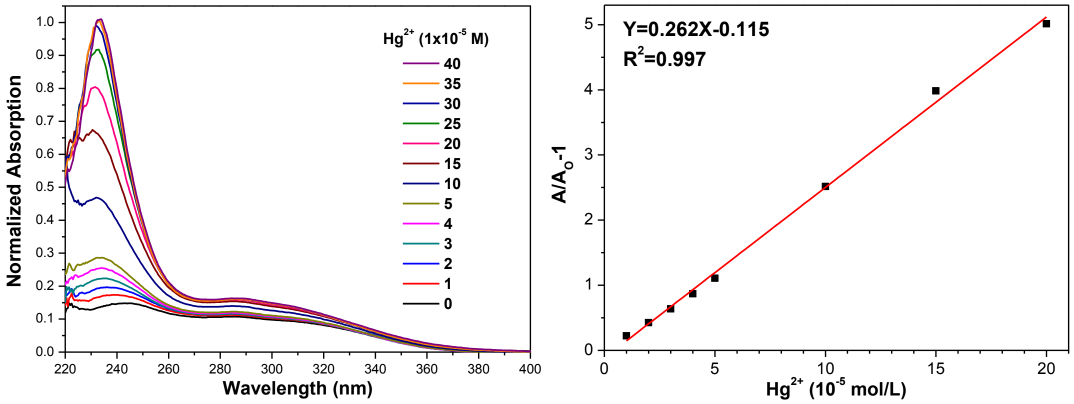

Figure S8. Absorption spectra of **TPE-S** (2.0 x 10^{-5} M) in THF in the presence of different amounts of Hg^{2+} and the plot of UV-Vis titration of **TPE-S** with Hg^{2+}.

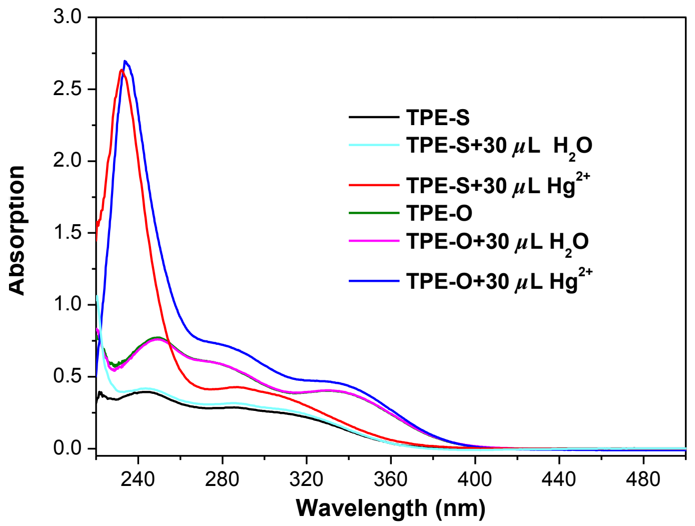

Figure S9. Absorption spectra of **TPE-O** and **TPE-S** (2.0×10^{-5} M) in THF in presence of 30 μL H_2O and Hg^{2+} (3.0×10^{-4} M).

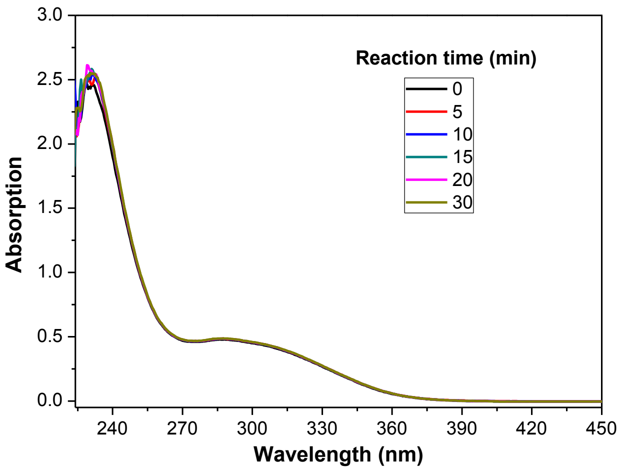

Figure S10. Absorption spectra of **TPE-S** (2.0×10^{-5} M) in THF in presence of Hg^{2+} (3.0×10^{-4} M) under different reaction time.

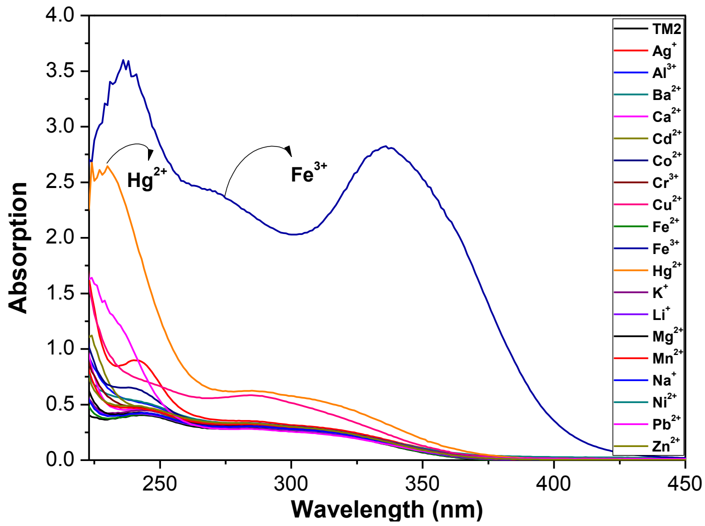

Figure S11. Absorption spectra of **TPE-S** (20 μM) in the presence of various metal ions (3×10^{-4} M).

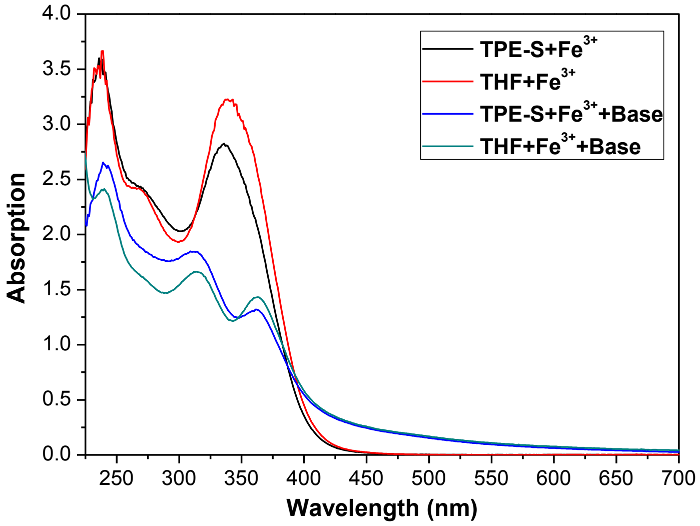

Figure S12. Absorption spectra of THF with/without **TPE-S** (2.0×10^{-5} M) in presence of Fe^{3+} (3.0×10^{-4} M).

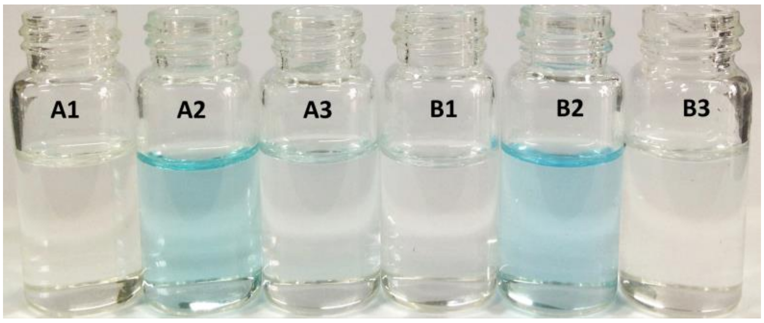

Figure S13. Photographs of (A1):**TPE-S** (2.0×10^{-5} M in THF); (B1): THF; (A2) and (B2): Co^{2+} (3×10^{-4} M) were added to **TPE-S** and THF; (A3) and (B3): added 0.1 mL H_2O following steps (A2) and (B2), respectively.

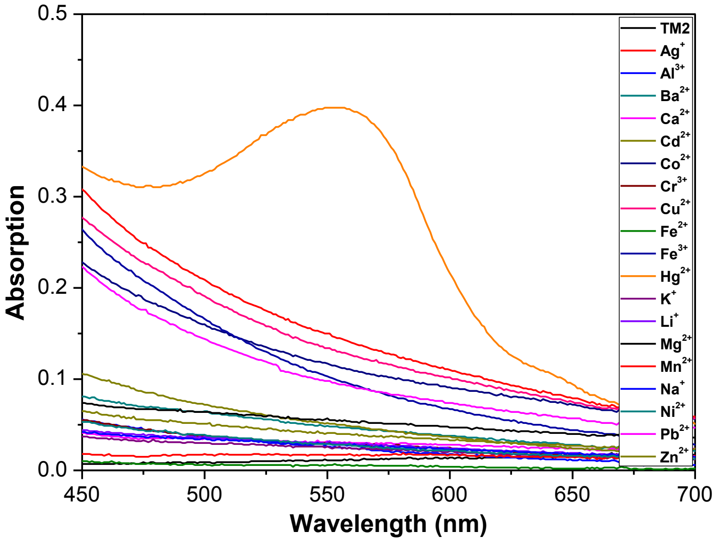

Figure S14. Absorption spectra of **TPE-S** (20 μM) in the presence of various metal ions (3×10^{-4} M), then added 30 equiv. of t-BuOK solution.

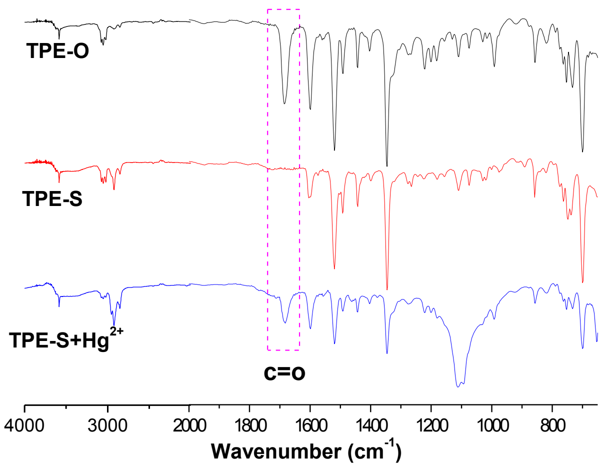

Figure S15. IR spectra of compounds **TPE-O**, **TPE-S** and the reaction product of **TPE-S** with Hg^{2+}.

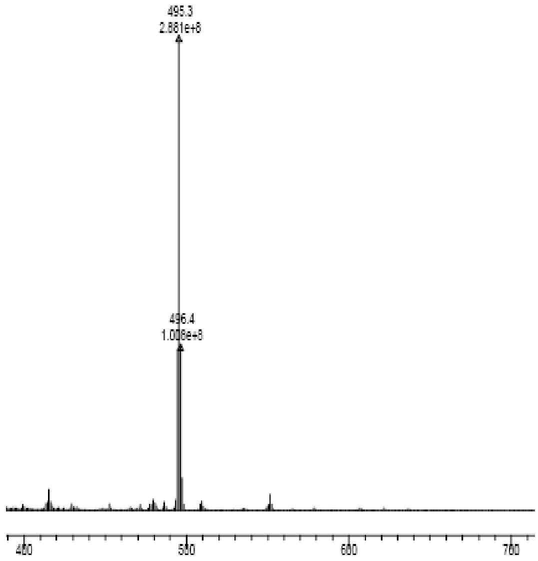

Figure S16. MS spectrum of the reaction product of **TPE-S** with Hg^{2+}, it was the same value as the formula mass of **TPE-O**.

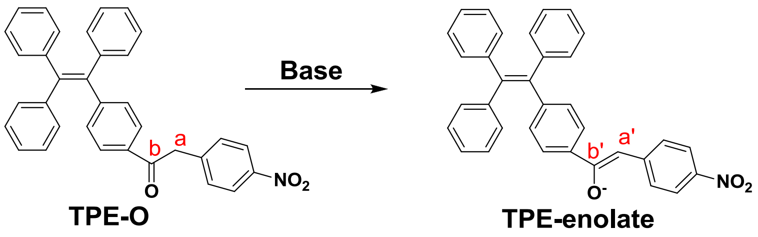

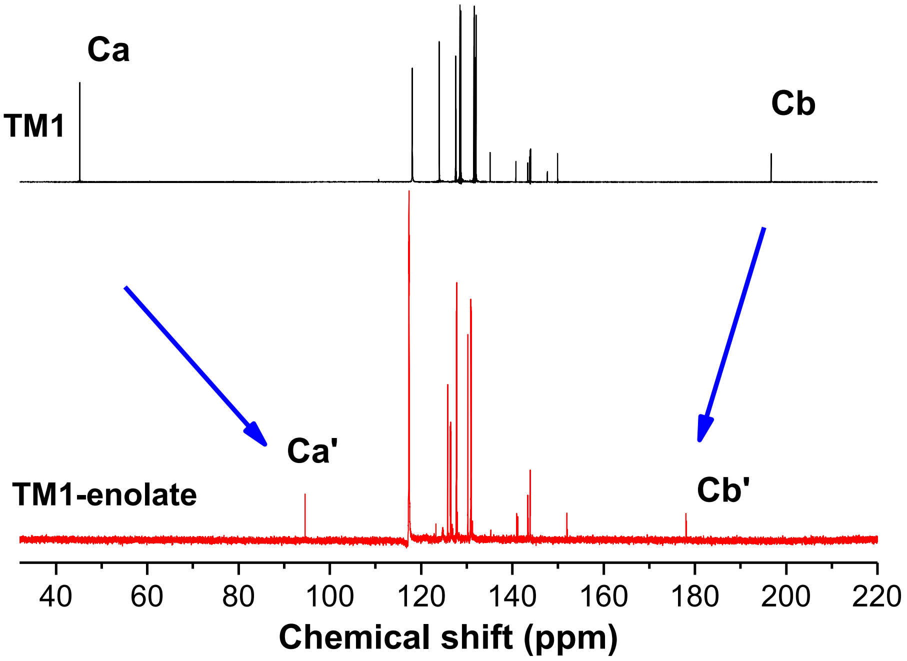

Figure S17. ^{13}C NMR spectra of compound **TPE-O** (in CD_3CN) before and after added *t*-BuOK.

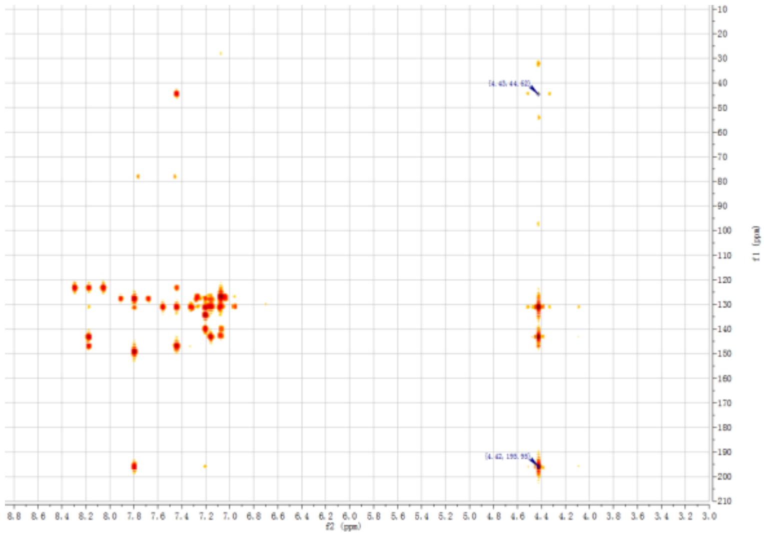

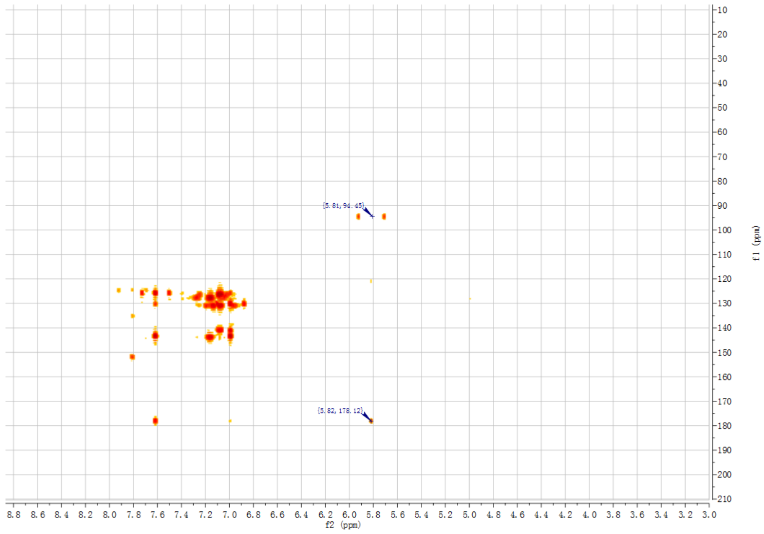

Figure S18. HMBC NMR spectra of **TPE-O** before (left) and after (right) added *t*-BuOK in CD_3CN.

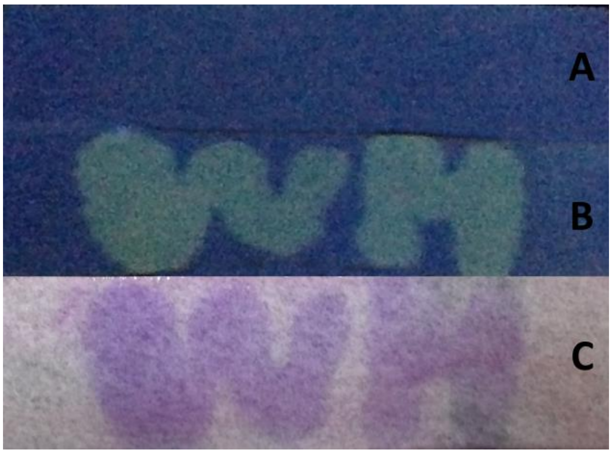

Figure S19. Photograph of fluorescence of **TPE-S** test strip (A), fluorescence and colorimetric response of **TPE-S** test strip with Hg^{2+} solution (1 x 10^{-3} mol/L) written on (B) and then dropped some t-BuOK (C).

EXPERIMENTAL SECTION

Materials and Instrumentation.

^{1}H and ^{13}C NMR spectroscopy study was conducted with a Varian Mercury 300 or Bruker ARX400 spectrometer using tetramethylsilane (TMS; δ = 0 ppm) as internal standard. HMBC spectra were measured on a 600 MHz Bruker Avance III NMR Spectrometer with a CryOProbe. EI-MS spectra were recorded with a Finnigan

PRACE mass spectrometer. Elemental analyses (EA) were performed by a CARLOERBA-1106 by a micro-elemental analyzer. The Fourier transform infrared (FTIR) spectra were recorded on a PerkinElmer-2 spectrometer in the region of 4000-400 cm^{-1}. UV/Vis spectra were obtained by using a Shimadzu UV-2550 spectrometer. Photoluminescence spectra were performed on a Hitachi F-4500 fluorescence spectrophotometer. Dichloromethane (DCM) was dried over and distilled from CaH_2. Tetrahydrofuran (THF) was dried over and distilled from K-Na alloy under an atmosphere of dry nitrogen. All other reagents were used as received without further purification.

Single crystal growth and analysis.

Single crystals were grown from the mixture solution of dichloromethane and hexane at room temperature.

The diffraction data for **TPE-O** were collected on an Agilent Technologies SuperNova Single Crystal Diffractometer that was equipped with graphite-monochromatized Mo Ka radiation (λ = 0.71073 Å) at 173 K. Empirical absorption corrections were applied to the intensities by using spherical harmonics, implemented in the SCALE3 ABSPACK scaling algorithm. The structure was solved by using the program SHELXS-97 and refined with the program SHELXL-97. All non hydrogen atoms were refined anisotropically. The hydrogen atoms of ligands were included in the structure-factor calculations at idealized positions by using a riding model and were refined isotropically. X-ray diffraction (XRD) intensity data are collected at 296 K for **TPE-S** on a Bruker Apex-II CCD diffractometer with

graphite monochromated Mo Kα radiation. Processing of the intensity data is conducted using the SANT and SADABS routines, and the structure and refinement are carried out using the SHELTL suite of X-ray programs (version 6.10) and OLEX2 (1.2.6). The crystal data are summarized in Table S1.

Synthesis and Characterization

TPE-O (2-(4-nitrophenyl)-1-(4-(1,2,2-triphenylvinyl)phenyl)ethanone): $SOCl_2$ (1 mL) was added to a solution of 2-(4-nitrophenyl)acetic acid (0.181 g, 1 mmol) in nitrobenzene (10 mL) and stirred at 65 ^{o}C overnight. Excess $SOCl_2$ was stripped off under vacuum at room temperature. Tetraphenylethylene (TPE) (0.332 g, 1 mmol) was added to the resulting solution with ice bath under nitrogen atmosphere, then $AlCl_3$ (0.133 g, 1 mmol) was added. After 4h, the reaction mixture was extracted with DCM for several times, the organic layer was combined, after evaporation of the DCM solvent, nitrobenzene was distilled off under vacuum. The crude product was purified by column chromatography using PE (petroleum ether)-EA (ethyl acetate) (10:1, V/V) as eluent to afford a yellow solid (0.316 g, 64 %). ^{1}H NMR (300 MHz, $CDCl_3$) δ (ppm): 8.21 (d, *J* = 8.4, 2H, ArH), 7.75 (d, *J* = 8.1, 2H, ArH), 7.40 (d, *J* = 8.1, 2H, ArH), 7.13 (m, 11H, ArH), 7.03 (m, 6H, ArH), 4.32 (s, 2H). ^{13}C NMR (100 MHz, $CDCl_3$) δ (ppm): 195.48, 149.69, 146.69, 142.96, 142.90, 142.82, 142.09, 139.53, 133.80, 131.69, 131.19, 131.14, 130.58, 127.85, 127.67, 126.93, 126.79, 123.60, 44.75. MS (EI), *m*/*z* [M^+]: 495.3, calcd: 495.2. Anal. calcd for $C_{34}H_{25}NO_3$: C 82.40, H 5.08, N 2.83; found: C 82.49, H 5.11, N 2.78.

TPE-S (2-(4-nitrobenzyl)-2-(4-(1,2,2-triphenylvinyl)phenyl)-1,3-dithiolane): Compound **TPE-O** (99 mg, 0.2 mmol) and 1, 2-ethanedithiol (0.025 mL, 0.3 mmol) were dissolved in dry dichloromethane (10 mL), then $BF_3 \cdot Et_2O$ (0.08 mL, 0.6 mmol) as the Lewis acid was added. After being stirred at 30 ℃ for 12 h, aqueous $NaHCO_3$ was added to the reaction mixture to adjust the pH value of 8-9. The resulting solution was extracted with DCM for several times, the organic layer was combined. The crude product was purified by column chromatography using PE -EA (10:1) as eluent to afford a light yellow solid (91 mg, 80 %). ^{1}H NMR (300 MHz, $CDCl_3$) δ (ppm): 8.00 (d, J = 8.1, 2H, ArH), 7.21 (m, 5H, ArH), 7.10 (br, 8H, ArH), 7.02 (br, 4H, ArH), 6.92 (m, 4H, ArH), 3.58 (s, 2H), 3.29 (m, 4H) ^{13}C NMR (100 MHz, $CDCl_3$) δ (ppm): 146.77, 144.55, 143.67, 143.39, 143.32, 143.02, 141.51, 141.29, 140.12, 131.31, 131.24, 131.19, 130.83, 127.63, 127.05, 126.66, 126.50, 73.76, 52.16, 39.13. MS (EI), m/z [M^+]: 571.3, calcd: 571.2. Anal. calcd for $C_{36}H_{29}NO_2S_2$: C 75.62, H 5.11, N 2.45; found: C 75.42, H 5.13, N 2.41.

TPE-1 (2-phenyl-1-(4-(1,2,2-triphenylvinyl)phenyl)ethanone): $AlCl_3$ (0.133 g, 1 mmol) was added to a solution of phenylacetyl chloride (0.155 g, 1 mmol) and TPE (0.332 g, 1 mmol) in dichloromethane (10 mL) with ice bath under nitrogen atmosphere, and stirred at room temperature overnight. The reaction mixture was extracted with DCM for several times, the organic layer was combined. After evaporation of the solvent, The crude product was purified by column chromatography using PE-DCM (2:1) as eluent to afford a colorless solid (0.365 g, 81 %). ^{1}H NMR (300 MHz, $CDCl_3$) δ (ppm): 7.77 (d, J = 8.7, 2H, ArH), 7.32 (m, 2H,

ArH), 7.22 (m, 2H, ArH), 7.12 (m, 12H, ArH), 7.02 (m, 6H, ArH), 4.21 (s, 2H). ^{13}C NMR (100 MHz, $CDCl_3$) δ (ppm): 197.14, 149.04, 143.11, 143.03, 142.95, 142.58, 139.73, 134.53, 134.29, 131.51, 131.22, 129.47, 128.53, 127.97, 127.83, 127.65, 126.88, 126.76, 126.73, 45.29. MS (EI), *m/z* [M^+]: 450.2, calcd: 450.2. Anal. calcd for $C_{34}H_{26}O$: C 90.63, H 5.82; found: C 90.39, H 5.71.

TPE-2 (2-benzyl-2-(4-(1,2,2-triphenylvinyl)phenyl)-1,3-dithiolane): Compound **TPE-1** (90 mg, 0.2 mmol) and 1, 2-ethanedithiol (0.025 mL, 0.3 mmol) were dissolved in dry dichloromethane (5 mL), then $BF_3 \cdot Et_2O$ (0.08 mL, 0.6 mmol) as the Lewis acid was added. After being stirred at 30 ℃ for 12 h, aqueous $NaHCO_3$ was added to the reaction mixture to adjust the pH value of 8-9. The resulting solution was extracted with DCM for several times, the organic layer was combined. The crude product was purified by column chromatography using PE -DCM (2:1) as eluent to afford a colorless solid (72 mg, 69 %). ^{1}H NMR (300 MHz, $CDCl_3$) δ (ppm): 7.22 (br, 2H, ArH), 7.14 (m, 14H, ArH), 7.02 (br, 4H, ArH), 6.92 (d, *J* = 8.1, 2H, ArH), 6.81 (m, 2H, ArH), 3.51 (s, 2H), 3.32 (m, 2H), 3.20 (m, 2H). ^{13}C NMR (100 MHz, $CDCl_3$) δ (ppm): 143.63, 143.50, 142.48, 142.34, 140.92, 140.37, 137.20, 131.52, 131.26, 130.74, 130.59, 130.50, 127.56, 127.32, 127.19, 126.69, 126.61, 126.49, 126.36, 74.65, 52.16, 38.86. MS (EI), *m/z* [M^+]: 526.2, calcd: 526.2. Anal. calcd for $C_{36}H_{30}S_2$: C 82.08, H 5.74; found: C 81.82, H 5.51.

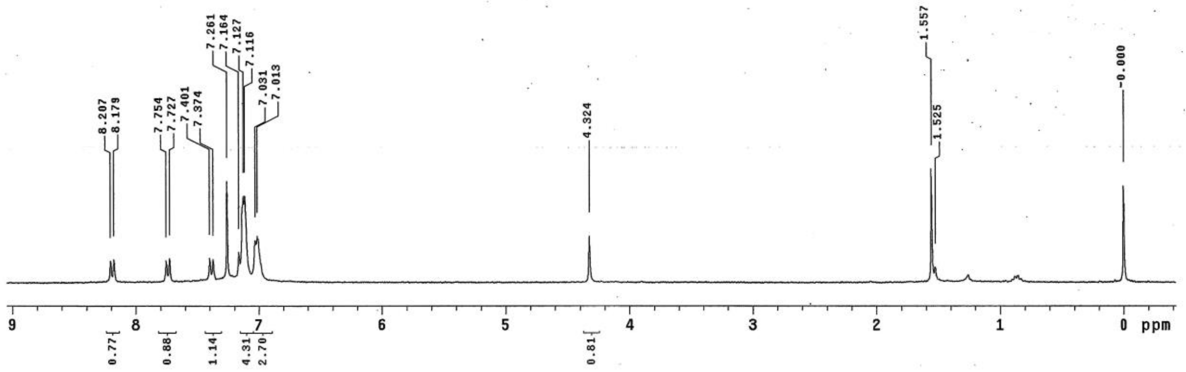

Figure S20. ^{1}H NMR of **TPE-O** in $CDCl_3$.

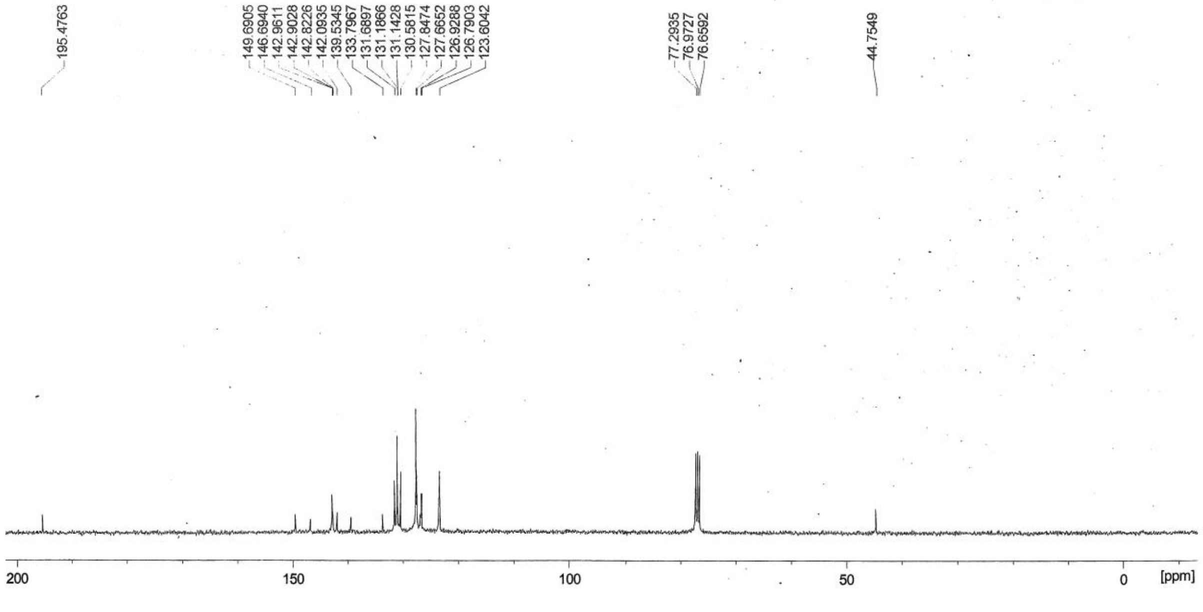

Figure S21. ^{13}C NMR of **TPE-O** in $CDCl_3$.

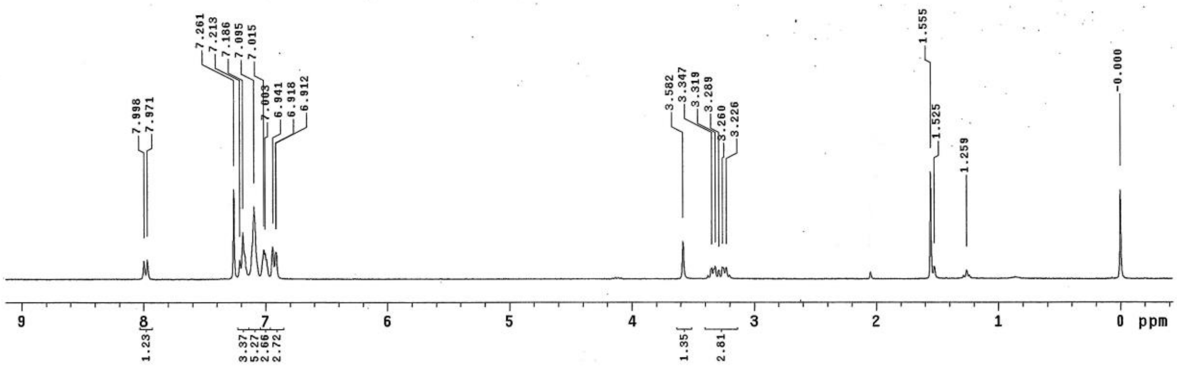

Figure S22. ^{1}H NMR of **TPE-S** in $CDCl_3$.

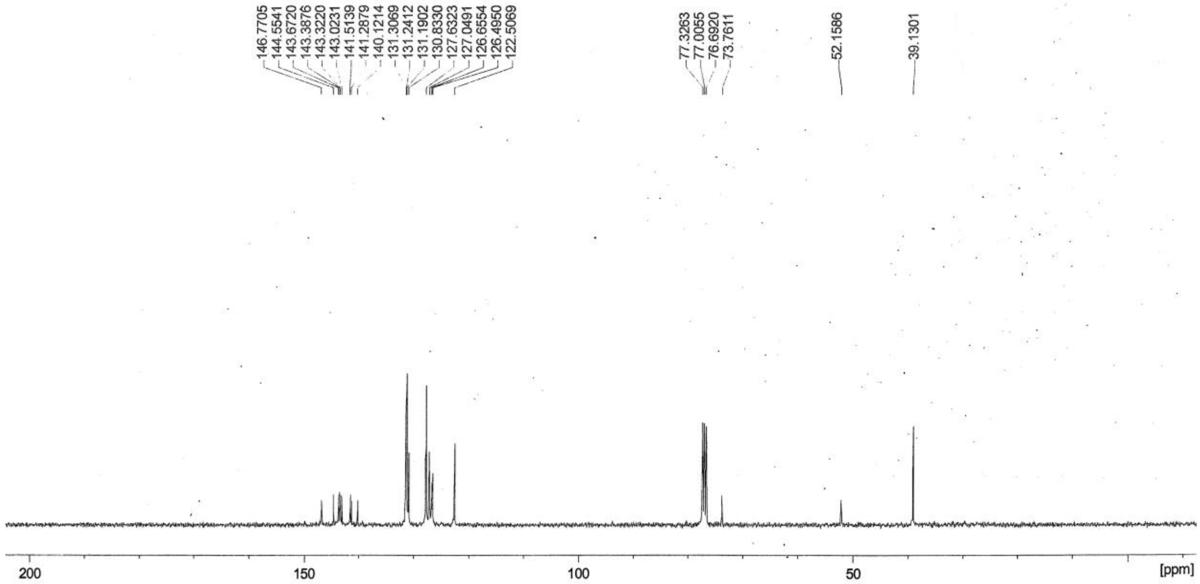

Figure S23. ^{13}C NMR of **TPE-S** in $CDCl_3$.

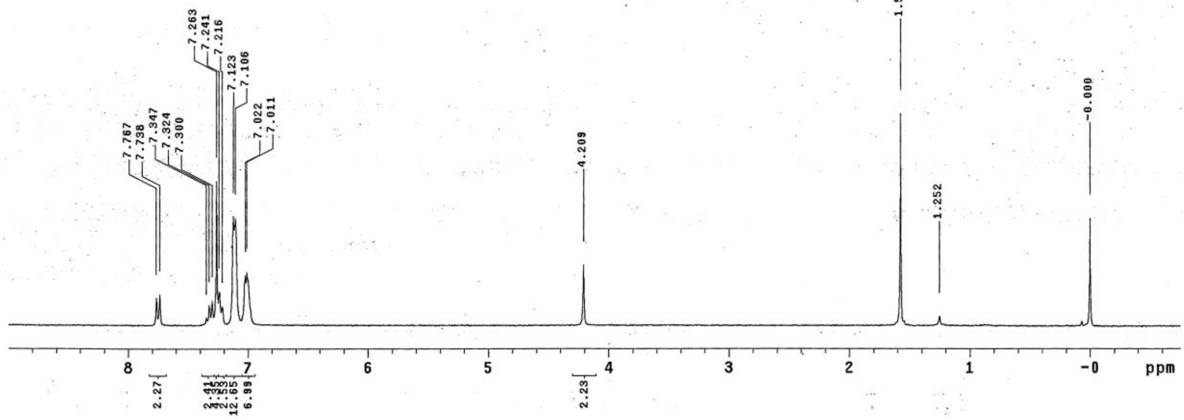

Figure S24. ^{1}H NMR of **TPE-1** in $CDCl_3$.

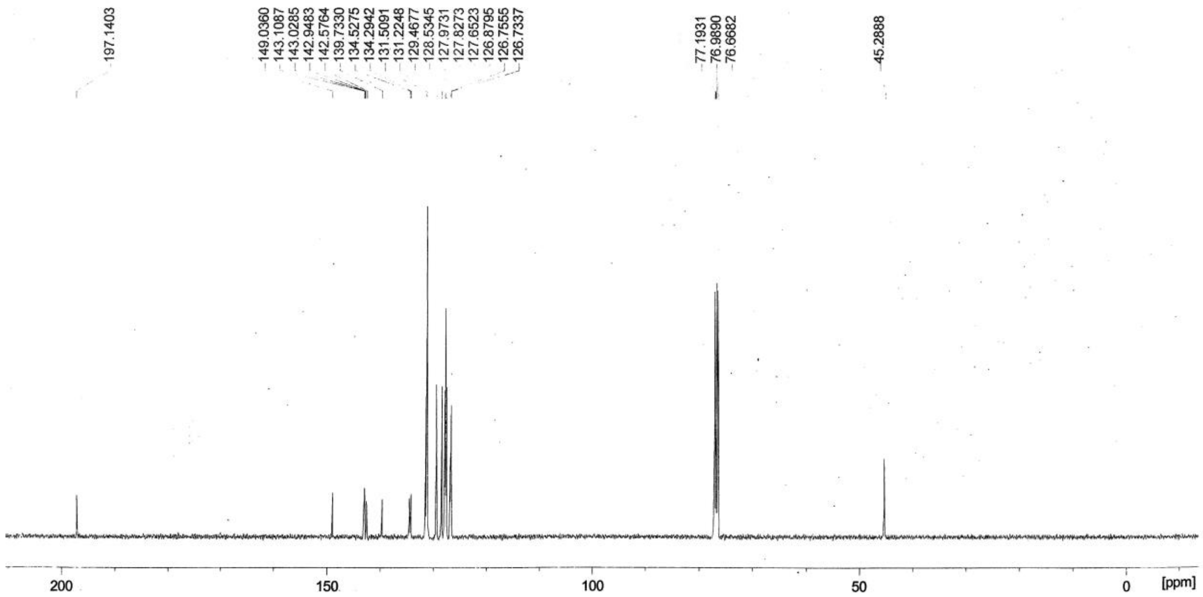

Figure S25. ^{13}C NMR of **TPE-1** in $CDCl_3$.

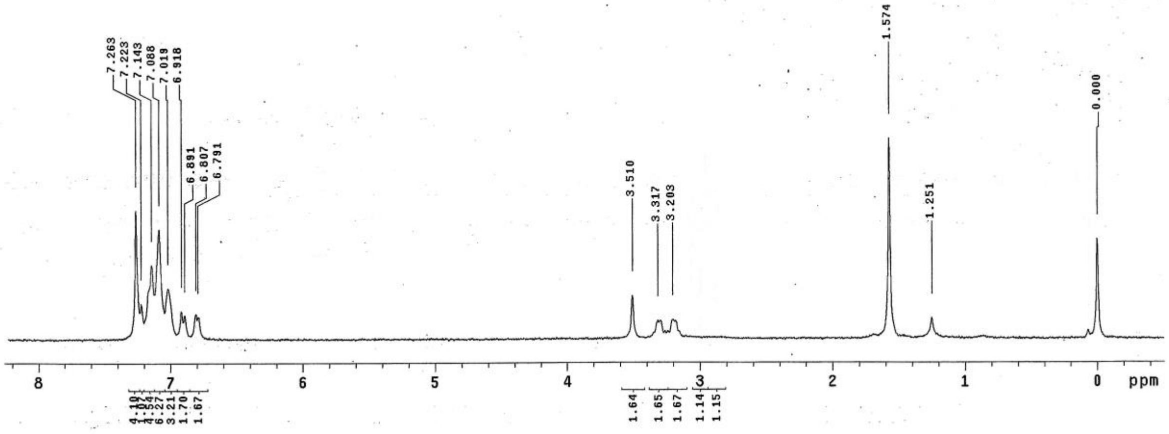

Figure S26. ^{1}H NMR of **TPE-2** in $CDCl_3$.

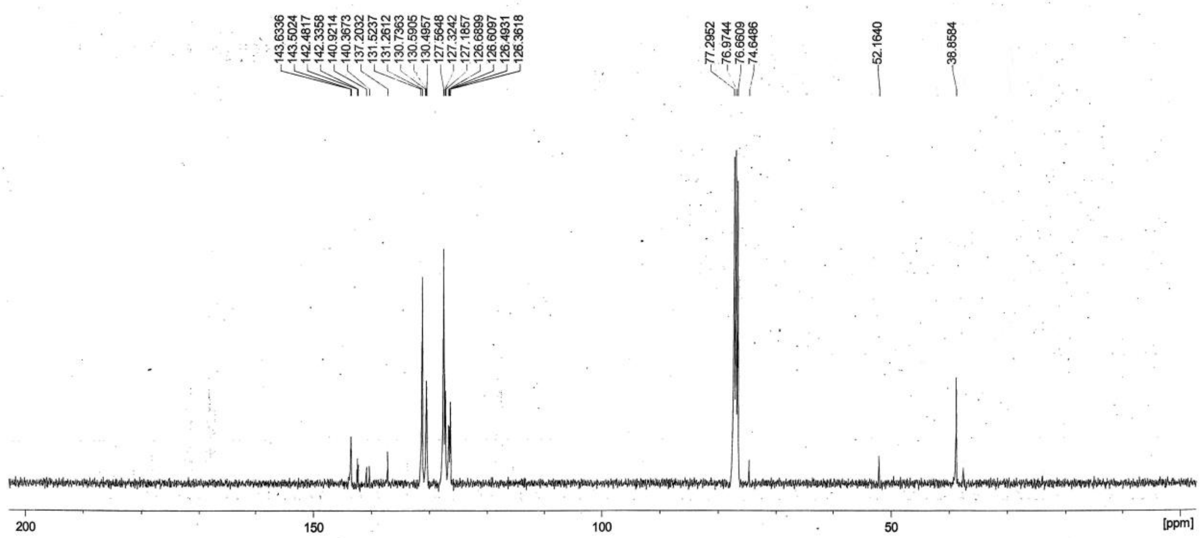

Figure S27. ^{13}C NMR of **TPE-2** in $CDCl_3$.
